# Morphology of the Jaw-Closing Musculature in the Common Wombat (*Vombatus ursinus*) Using Digital Dissection and Magnetic Resonance Imaging

**DOI:** 10.1371/journal.pone.0117730

**Published:** 2015-02-23

**Authors:** Alana C. Sharp, Peter W. Trusler

**Affiliations:** School of Earth, Atmosphere and Environment, Monash University, Clayton, Victoria, Australia; Max Planck Institute for Evolutionary Anthropology, GERMANY

## Abstract

Wombats are unique among marsupials in having one pair of upper incisors, and hypsodont molars for processing tough, abrasive vegetation. Of the three extant species, the most abundant, the common wombat (*Vombatus ursinus*), has had the least attention in terms of masticatory muscle morphology, and has never been thoroughly described. Using MRI and digital dissection to compliment traditional gross dissections, the major jaw adductor muscles, the masseter, temporalis and pterygoids, were described. The masseter and medial pterygoid muscles are greatly enlarged compared to other marsupials. This, in combination with the distinctive form and function of the dentition, most likely facilitates processing a tough, abrasive diet. The broad, flat skull and large masticatory muscles are well suited to generate a very high bite force. MRI scans allow more detail of the muscle morphology to be observed and the technique of digital dissections greatly enhances the knowledge obtained from gross dissections.

## Introduction

Many morphological characteristics reflect the grazing nature of wombats. Like other members of the order Diprotodontia, wombats have only two lower incisors, but they are unique among marsupials in having only one pair of upper incisors. The cheek teeth, one premolar and four molars on each side of the jaw, have a flat surface, and are rootless, allowing them to grow continuously throughout the animal’s life. When feeding, the lower jaw moves sideways and upwards so that the blades of the molars meet to cut the grass stems [1,2]. They have a bilateral chewing function where only the muscles on the working side activate during the power stroke [3,4]. This is an efficient way of processing the course, abrasive grasses that are the wombat’s dominant food [1].

The common wombat, *Vombatus ursinus*, is the largest of the three species of wombats, weighing between 23 and 38 kg, and having a body length from 850 to 1140 mm [5,6]. The northern hairy-nosed wombat (*Lasiorhinus krefftii*) is about the same size as *V*. *ursinus*, but the southern hairy-nosed wombat (*Lasiorhinus latifrons*) is slightly smaller at 22–25 kg and 850 mm in length [1]. Of these species, *V*. *ursinus* is the most abundant. However, it has had the least attention in terms of masticatory muscle morphology, and has never been thoroughly described. In addition, conclusions have been drawn for both *V*. *ursinus* and *L*. *latifrons* without any published illustrations or descriptions of the muscle anatomy for *V*. *ursinus*. While these genera are considered to be anatomically similar, they live in very different environments and have different diets [1]. Thus, any conclusions based on soft-tissue morphology and function of *L*. *latifrons*, may not apply for *V*. *ursinus*.

Murray [7] and Crompton et al. [4] detailed the anatomical features of the jaw muscles for *L*. *latifrons*. However, there is little consistency in the terminology and measurements of muscle weights between the two studies. The designation of the three major masticatory groups, *m*. *temporalis*, *m*. *masseter* and *m*. *pterygoideus*, needs detailed reassessment and possible redefining. Detailed studies of the muscle structures in macropodoidea, and a wide range of placental herbivores, indicates that the masticatory muscle system is more complex than those typically documented for carnivores [8–11]. The added complexity arises as a result of incomplete fascia that give rise to interdigitating muscle bands within and between masticatory groups. Uncertainties typically concern the masseter and temporalis and the number of components into which each muscle divides. Confusion may be compounded by the misidentification of complete and partial divisions and application of nomenclature that may not reflect homologies.

A possible cause of this uncertainty is the invasive and destructive nature of dissections: superficial structures are removed and weighed first, while deeper structures desiccate and lose mass over time; blunt dissection between muscle layers often means that the spatial relationships are difficult to define accurately; and, to access very deep and confined structures, like the pterygoid muscles, removing the zygomatic arch or coronoid process may be necessary. For these reasons, we employ an alternative method, “digital dissection”, to compliment gross dissections of *V*. *ursinus* in order to obtain a greater understanding of the muscle anatomy.

Techniques for imaging muscle anatomy have become considerably more varied over the last few years [12–17]. Computed tomography (CT) scanning, magnetic resonance imaging (MRI) and contrast enhanced CT imaging using Iodine Potassium Iodide (I_2_KI) and Phosphotungstic acid (PTA), have enabled the visualisation of soft tissues to compliment more traditional techniques such as dissection. These methods allow us to achieve greater understanding of the spatial arrangement, muscle fibre architecture and complexity of soft tissues, as well as providing additional means to measure volume, fibre direction and length, cross sectional area and muscle force.

The following three-dimensional (3D) reconstruction and description of the jaw musculature of *V*. *ursinus* was motivated by the lack of detailed anatomical data on this species. Digital, and gross dissections of the jaw adductor muscles were undertaken with the aims to: 1) provide a detailed anatomical description of the jaw adductor muscles that function to close the jaw; 2) gather quantitative data on relative muscle size; 3) map the area of the muscle origins and insertions; and, 4) compare the jaw muscle morphology of *V*. *ursinus* to that of *L*. *latifrons* to provide data for further studies given the importance of wombats in comparative anatomy and palaeontology of marsupials. The 3D model of the skull and muscles was constructed using CT and MRI data to visualise the spatial arrangement of the muscles, and to measure the volume of each muscle as an estimate of absolute and relative muscle size. The 3D model is available as an interactive 3D Adobe Portable Document Format (3D PDF) file (S1 Fig), viewable on the freely available Adobe Reader [18,19].

## Materials and Methods

### Nomenclature

For marsupial taxa, most authors agree on the terminology for the masseter layers as superficial and deep, with the inclusion of the zygomaticomandibularis as the deepest layer of the group [7,9,10,20]. However, there are some variations. For *L*. *latifrons*, Crompton et al. [4] described two layers of the superficial masseter (termed internal and external) and a deep masseter, but does not describe the zygomaticomandibularis. The internal superficial masseter is described as originating on the ventral surface of the zygomatic arch, and the deep masseter as originating from the medial surface of the zygomatic arch. Based on their origins, these muscles could have been named the deep masseter and zygomaticomandibularis respectively, which are more commonly used terms. Other variations include multiple layers of the superficial masseter in Macropodoidea [9,11], and the addition of an intermediate masseter [11]. Here, the masseter will be referred to as having four layers (superficial, intermediate, deep and zygomaticomandibularis) based on their origins. Each of these muscles may also include multiple layers if partial fascia are present.

There is less consistency in the literature for the temporalis muscle group. Most agree on three components, but the names designated for each vary. Crompton et al. [4] described the temporalis in *L*. *latifrons* as having a posterior layer and a deeper anterior layer. The deep layer has also been described as inferior in *Phascolarctos cinereus* [20] and middle in *Macropus giganteus* [9], but is generally referred to as deep in most other taxa [10,11]. To avoid confusion, we refer to this layer as ‘deep’. The fan shaped posterior layer is also referred to as medial [11], superior [20] and superficial [10]. We will use ‘posterior’, based on the posterior location of the origin within the temporal fossa. The third layer is often described as a division of the posterior temporalis (*pars zygomatica* by Turnbull, 1970) or the superficial lateral layer based on its origin on the zygomatic arch. Here, we describe the superficial lateral layer as separate from the posterior layer.

Here, the pterygoid muscles are referred to as medial and lateral, rather than internal and external as in Turnbull [10], as this is the most common use in Vombatidae and most other marsupial families [4,7,9,11,20]. The medial pterygoid (referred to as *m*. *pterygoideus internus* by Turnbull [10]) is often described as having two parts: a deep and superficial part. Inconsistency in the nomenclature has made the distinction between these two parts unclear. Abbie [21] and Crompton et al. [4] define the deep medial pterygoid as passing vertically downwards from its origin on the ventral edge of the pterygoid bone to its insertion on the medial edge of the inflected angle of the mandible, and the superficial part as originating from the lateral plate of the pterygoid process of the sphenoid and inserting on the walls and floor of the pterygoid fossa. Tomo et al. [9], and subsequently Warburton [11], referred to these two parts in the opposite way for Macropodidae. Here we have adopted the terminology of Abbie [21] and Crompton et al. [4], because the “deep” portion is closer to the midline of the skull.

### Data Acquisition and Processing

A research permit was granted from the Victorian Department of Sustainability and Environment to receive and retain specimens of wildlife found dead from natural or accidental causes (Flora and Fauna Permit number 10005574). The skulls of two adult common wombats (*Vombatus ursinus*), one male and one female, were dissected after collection from the Australian Wildlife Health Centre at Healesville Sanctuary. One deceased adult female common wombat was also collected from Museum Victoria. No animals were euthanized for this study; all specimens died of natural or accidental causes. Once obtained, the specimens were frozen, and then thawed when scanning could be scheduled. To ensure minimal decomposition, the specimens were subsequently refrozen and thawed again when it was not practical to conduct the dissection immediately after the scan. While these steps could result in some alteration of soft tissue, it was considered preferable to longer-term deterioration of fresh tissue due to time delays. The muscles of mastication were dissected on both sides of each specimen.

Some variation to the method and sequence of dissection was undertaken between specimens to record and collect all the necessary data. To weigh the muscles, careful attention was paid to removing the entirety of each muscle from both the origin and insertion. The wet muscles were immediately weighed following removal. The mean weight between right and left sides was recorded and the percentage of the total mean mass of each muscle was determined. To view the muscle attachment areas for the pterygoids, the muscles were sectioned halfway along their length, or at the widest part of their bellies, and the mandible removed. The two portions of each muscle were then removed and weighed together. In the case where muscles fused at deeper layers to form a continuous muscle mass, such as the interdigitated nature of the masseter, the muscle bundles were divided by blunt dissection along fibre planes, in order to record the weight of each, a method used by Davison and Young [20]. As the dissection proceeded from superficial to deep layers, photographs, line drawings, and markings on dry skulls were used to record muscle components, including fibre direction and origins and insertions.

### Model Construction

**Fig 1:**
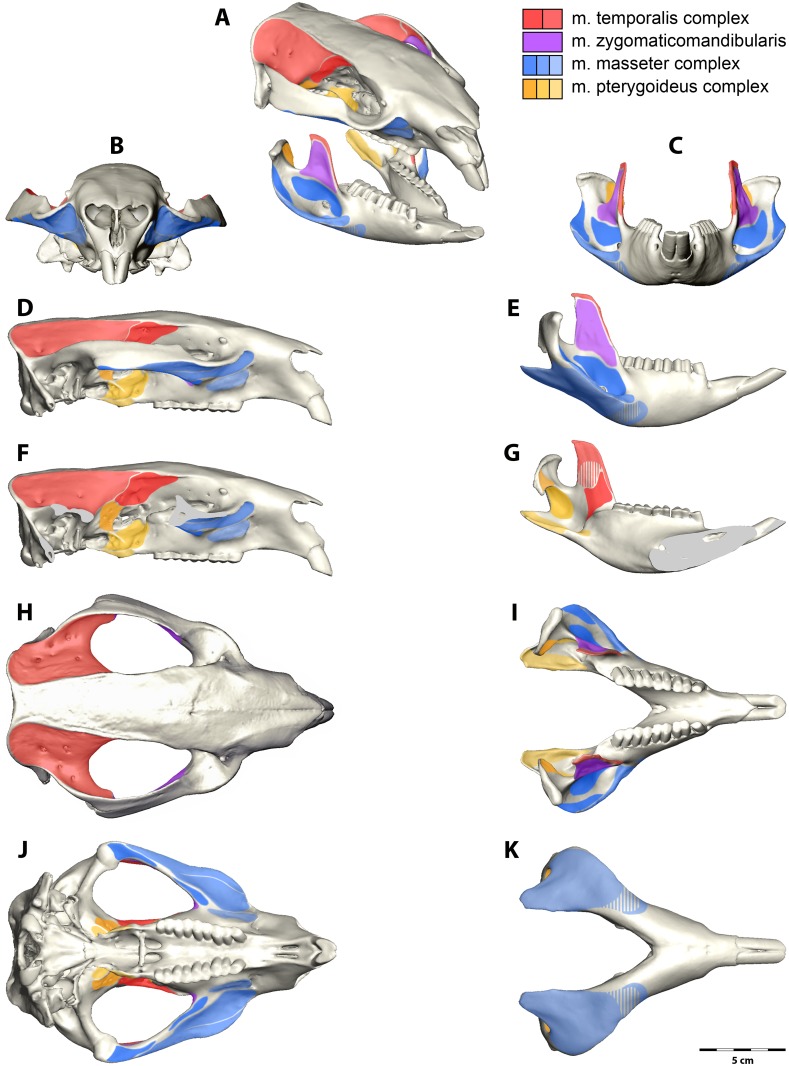
Jaw muscle attachment areas for the common wombat, *Vombatus ursinus*. (A) Oblique view, (B, C) anterior view, (D, E, F. G) lateral view, (H, I) dorsal view and (J, K) ventral view. View (F) shows the cranium in lateral view with the zygomatic arch removed for visualization of the pterygoid muscle origins. View (G) shows the medial view of the mandible with pterygoid and temporalis insertion areas. Areas where the muscle is not strongly attached are marked with broken lines. The legend indicates the colour for each muscle group.

**Fig 2:**
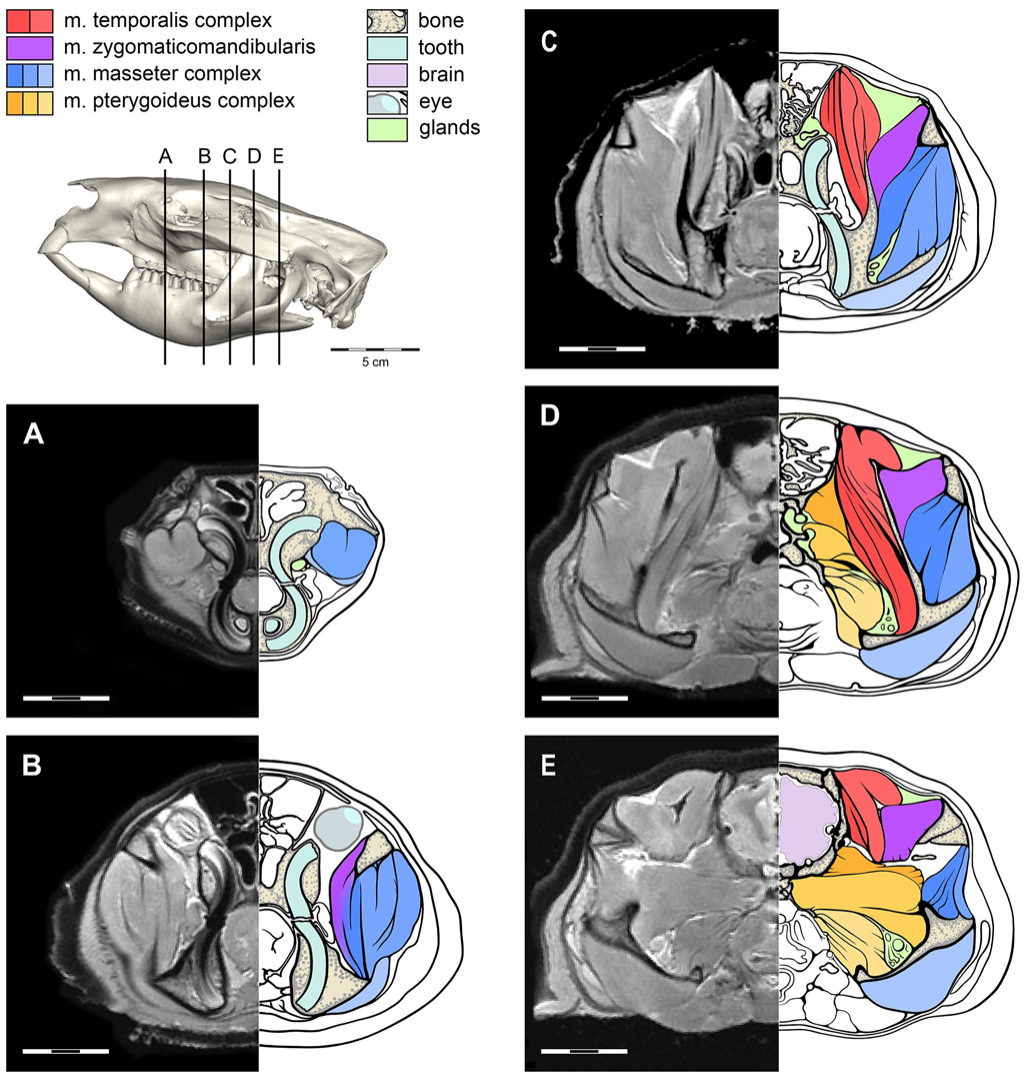
The arrangement of the jaw muscles as visualized from the MRI. The legend indicates the colour for each muscle group as well as non-muscle anatomy. The location for each MRI slice is indicated on the lateral view of the skull.

One adult female specimen was scanned by X-ray computed tomography (CT) and *magnetic resonance imaging* (MRI) at the University of Melbourne Veterinary Hospital for construction of a 3D model. The MRI was set to proton density (PD)-weighted and scanned with a 3.0 mm slice thickness and 0.625/0.625 pixel spacing in the frontal plane, and 2.5 mm slice thickness and 0.55/0.55 pixels for the horizontal and sagittal planes. The CT scans have a 0.6 mm slice thickness, 0.3 mm interslice distance and 0.3125/0.3125 pixels. The CT (S1 Movie) and MRI (S2 Movie) data are available as movie files in the supplementary information. The CT and MRI data were imported into the image visualisation and processing software program Avizo (Visualization Science Group), in which manual segmentation using the paintbrush tool (the process of selection and isolation of the structure of interest) was used to isolate the craniodental morphology and individual muscles into separate materials. Muscle origin and insertion sites (Fig. 1) for *V*. *ursinus* were identified for each muscle based on dissections and MRI scans (Fig. 2). Each muscle was selected first in the coronal plane and then edited in the remaining two planes for biological accuracy (sagittal and horizontal plane). When each muscle, and the cranium and mandible were assigned to separate materials, a 3D surface model was produced using the Avizo “SurfaceGen” module, which constructs a mesh of triangles and nodes that represent the continuum structure. The volume for each muscle was obtained from the 3D model using the Avizo “MaterialStatistics” module, and the percentage of each muscle of the total volume was determined.

### 3D PDF

The digital record of the cranium, mandible and each muscle were exported from Avizo as separate 3D surface models to create the 3D PDF, following a similar approach to Lautenschlager [19]. Each surface was down-sampled in Avizo to reduce the number of surface triangles, thereby producing smaller files to export. The models were then exported as Wavefront (*.obj) generic 3D files. The surfaces were imported to Adobe 3D Reviewer (available in Adobe Acrobat 9 Pro Extended) and converted to a single Universal 3D (*.u3d) file. The U3D file was then imbedded in the PDF document using Adobe Acrobat 9 Pro. The background was set to “white”, and the lighting was set to “headlamp”. Standard anatomical views (lateral, anterior, posterior, dorsal and ventral) were also assigned. The 3D PDF can be viewed using Adobe Reader by clicking on S1 Fig.

## Results

The total weights for each muscle group, for each *Vombatus ursinus* specimen dissected, are compared to the data for *Lasiorhinus latifrons* provided by Crompton et al. [4] (Table 1).

**Table 1:** Weight and percentage of the total weight of the adductor muscles in *Vombatus ursinus* and *Lasiorhinus latifrons* collected from dissections.

Species	*Vombatus ursinus*								*Lasiorhinus latifrons*	
	**Specimen 1**		**Specimen 2**		**Specimen 3**					
	**Weight**	**%**	**Weight**	**%**	**Weight**	**%**	**Mean Weight**	**Mean %**	**Weight**	**%**
**Masseter**	101.9	50.7	82.8	59.9	92.0	58.5	92.2	56.4	69.7	51.8
**Temporalis**	68.2	33.9	37.0	26.8	46.2	29.3	50.5	30.0	42.1	31.3
**Pterygoid**	31.0	15.4	18.4	13.3	19.3	12.2	22.9	13.6	22.7	16.9
**Total**	201.1	100	138.2	100	157.5	100	165.6	100	134.5	100

Specimen 1 is male, and specimen 2 and 3 are female. *L*. *latifrons* data from Crompton et al. [4], sex unknown.

### Muscle morphology

#### Masseter Complex

**Fig 3:**
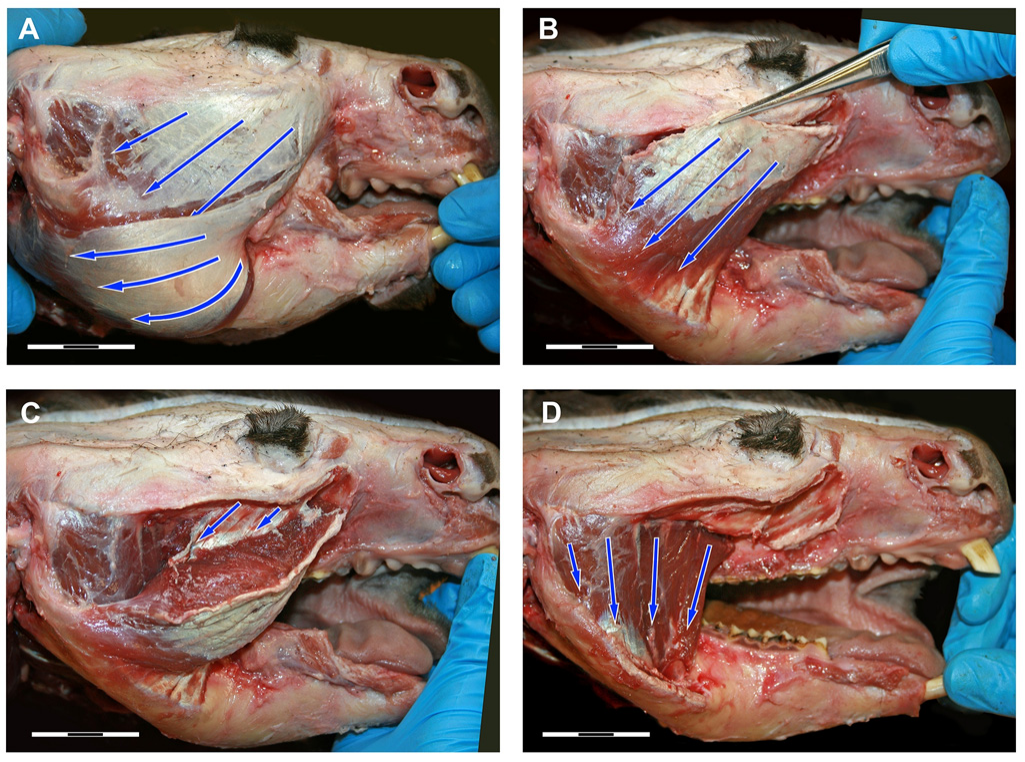
Dissection photos of the masseter muscle in lateral view showing fibre direction. (A) The most superficial layer of the masseter showing the fibre direction of the internal and external superficial masseter; (B) External superficial masseter removed to reveal the internal superficial masseter; (C) Part of the internal superficial masseter pulled back to reveal the fascia that partially separates the internal superficial masseter at its origin; (D) Internal and external superficial masseter removed to reveal the posterior and anterior deep masseter.

**Fig 4:**
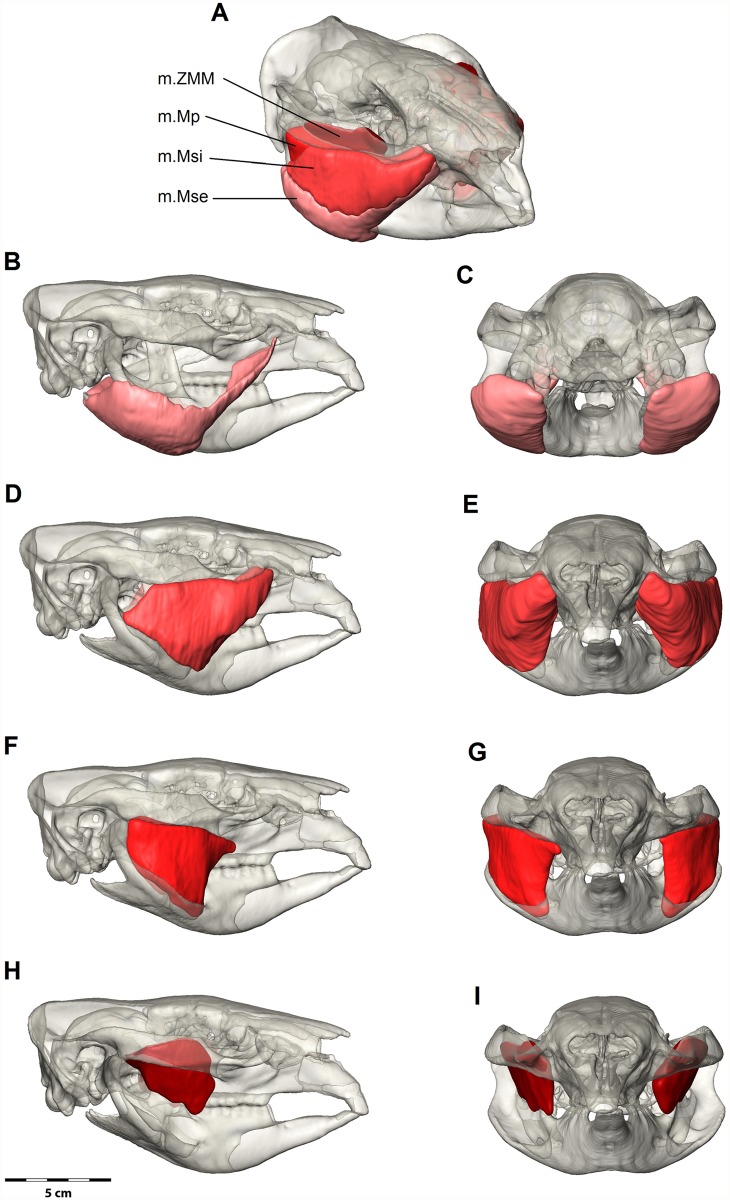
Digital dissection of the masseter muscle group. (A) Oblique view with all masseter muscles; (B, C) External superficial masseter (m.Mse) in lateral and posterior views; (D, E) Internal superficial masseter (m.Msi) in lateral and anterior views; (F, G) Deep masseter (m.Mp); (H, I) Zygomaticomandibularis (m.ZMM). Bone is at 70% transparency.

The masseter is a large and complex muscle in the wombat (Figs. 3–4). It contributes over half of the total masticatory muscle mass and consists of four major parts with each major muscle belly interdigitating with its neighbour. The four major parts can be further separated into a total of six muscles bundles, some of which are strongly attached by tendinous aponeuroses at their origins and/or insertions. These tendinous attachments broaden to form expansive fascia over portions of the muscle bellies, but do not entirely separate them. Thus, the masseter comprises interleaved muscle sheets with varying fibre directions. This interdigitated nature of the masseter complex is well reflected in the MRI scans (Fig. 2). In Fig. 2B, the dark bands that represent the fascia between muscle bundles are discontinuous along the length of each muscle.

*M*. *masseter superficialis externus* (m.Mse) has a very small, strongly tendinous origin on the maxillojugal region of the zygomatic arch, located anterior to the orbit and the premolar (Figs. 1 and 4). This constitutes the anteroventral portion of a sharply defined fossa across the maxillojugal suture, immediately posterolateral to the infraorbital foramen. This origin is strongly bound by a prominent aponeurosis that fans out in a posteroventral direction and covers approximately three quarters of the muscle surface. The fibre bundles form a gradually radiating structure that inserts from the ventral edge of the masseteric crest to the base of the ascending articular process and covers the entire posteroventral surface of the inflected mandibular angle. The anterior half of this radiating arrangement forms a distinctive ‘rolled under’ margin to the masseter complex; termed *pars reflexa* in *L*. *latifrons* by Murray (1998). The posterior portion (not covered by the aponeurosis) is extensive; with a weak fleshy insertion over the entire posterior surface of the mandibular angle, as described above, and tendinously bound along the posterior extent of the mandible edge.

*M*. *masseter superficialis internus* (m.Msi) originates in two clearly delimited regions on the anterior two thirds of the jugal. The first area extends across the ventrolateral surface of the jugal from the anterior orbital rim. A strong superficial aponeurosis runs along the lateral edge of the zygomatic arch and the parallel muscle fibres of the internal superficial masseter pass posteroventrally at approximately 45° to the horizontal (Fig. 3B). The second, deeper origin fills the excavated maxillojugal region located under the orbit. An incomplete fascia separates the two muscle bundles of the internal superficial masseter at the origin (Fig. 3C). The distinction between the internal and external superficial masseter layers at this anterior point is lost with the rolling of the *pars reflexa*. The internal superficial masseter is thickest at this point and thins posteriorly to insert on the medial edge of the masseteric crest. The internal superficial masseter inserts strongly by a tendinous aponeurosis to the anterior edge of the masseteric fossa, along the masseteric crest and dorsally to the widest point of the masseteric crest.

*M*. *masseter profundus* (m.Mp) originates from two regions and comprise two separate bundles. A muscle bundle that originates from the ventral edge of the posterior half of the zygomatic arch, runs ventrally and inserts in the masseteric fossa, termed posterior deep masseter (Fig. 3D). This muscle bundle is only partly covered by the intermediate masseter and is visible in lateral view. A second bundle has an anterior origin (anterior deep masseter) along the thin ventral edge of the zygomatic arch. It is clearly divided by a strong fascia from the internal superficial masseter, and separating the two muscles is glandular and vascular tissue emanating from the masseteric foramen. The anterior deep masseter inserts on the lateral surface of the ascending ramus.

*M*. *zygomaticomandibularis* (m.ZMM) originates from the medial surface of the zygomatic arch (Figs. 1, 2 and 4). The posterior area of origin is inferior to the *m*. *temporalis lateralis* and delimited by the squamosal-jugal suture. This posterior part of the muscle is intimately united with the lateral temporalis and separates from it anteriorly, replacing the temporalis at the anterior of the zygomatic arch. Thus the origin covers almost the entire medial surface of the zygomatic arch to an anterior limit immediately ventral and posterior to the postorbital ligament and the frontal process of the jugal. From its origin the muscle fibres converge toward their insertion. The anterior part is oriented at 30–40° to the horizontal and inserts along the anterior border of the ascending ramus. The posterior portions run ventrally and inserts to the lateral surface of the ascending ramus superior to the insertion of the deep masseter. Numerous fascia partially separate the muscle at its insertion on the ascending ramus so that the muscle is not easily removed during dissection.

#### Temporalis Complex

**Fig 5:**
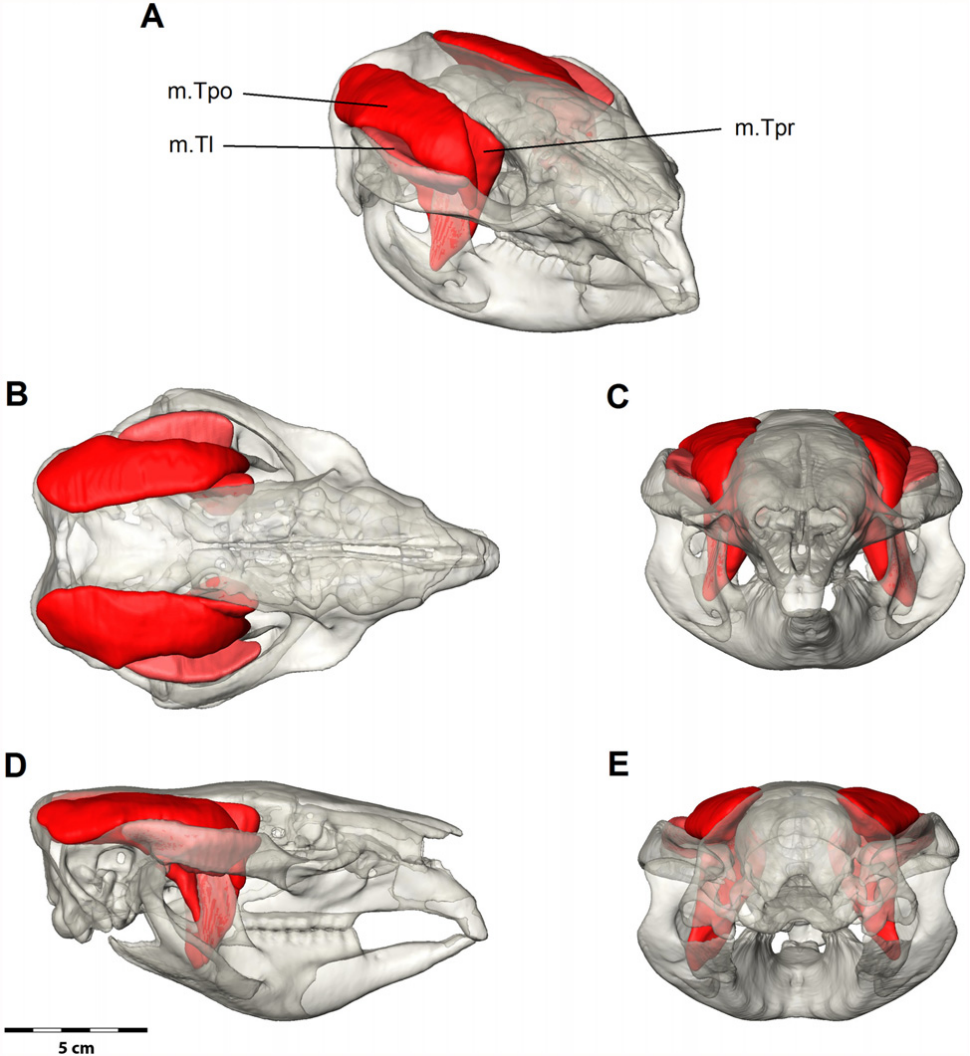
Digital dissection of the temporalis muscle group. (A) Oblique, (B) dorsal, (C) anterior, (D) lateral and (E) posterior views. m.Tpr = deep temporalis, m.Tl = lateral temporalis, m.Tpo = posterior temporalis. Bone is at 70% transparency.

The temporalis complex (Fig. 5) is formed by three main components: the lateral, posterior and deep parts. It is smaller than the masseter complex but larger than the pterygoid complex (Table 1). A strong aponeurosis covers the temporalis and fuses to the postorbital ligament. A thin superficial fascia covers the aponeurosis and runs over the zygomatic arch and the masseter.

*M*. *temporalis lateralis* (m.Tl), the smaller lateral bundle of the temporalis, originates from the posterolateral portion of the nuchal crest and curves anteromedially and ventrally to form a cleft with those of the *m*. *temporalis posterior*. The area of origin extends anteriorly on the dorsomedial surface of the zygomatic arch, uniting with the origin of the *m*. *zygomaticomandibularis*. The boundary between the *m*. *temporalis lateralis* and the *m*. *zygomaticomandibularis* on the medial surface of the zygomatic arch is not indicated by fascia and the respective muscles appear intimately fused. The *m*. *temporalis lateralis* continues ventrally to insert on the lateral tip of the coronoid process.

*M*. *temporalis posterior* (m.Tpo) is the largest of the three temporal components and has an extensive origin on the frontal and parietal bones, covering the majority of the dorsal surface of the temporal fossa. Some fibres (and blood vessels) attach deep into the small foramina that punctuate the bone surface of the temporal fossa forming pits. The muscle is bordered posteriorly by the occipital crest and dorsally by the midsagittal plane. The muscle is narrowly pennate with fibres converging anteriorly to join a strong internal aponeurosis that inserts along the tip of the coronoid process. As the muscle passes forwards, it curves ventrally to form a sagittally aligned cleft between the *m*. *temporalis lateralis* and *m*. *zygomaticomandibularis*. It is this cleft that confers a strongly pennate appearance to the temporal system. The cleft is occupied by a very large lacrimal gland that extends under the postorbital ligament to the posterior surface of the periorbita. The *m*. *temporalis posterior* continues ventrally to form a strong tendinous insertion along the anterior edge of the coronoid process.

*M*. *temporalis profundus* (m.Tpr) is a bundle of several components originating on the lateral surface of the cranium immediately posterior to the zygomatic process of the frontal. The muscle appears striated in the MRI due to many separating fascia (Fig. 2C and 2D). The origin runs posteriorly along the temporal line to the coronal suture and faces anteroventrally, but the ventral boarder does not include the lateral face of the alisphenoid. The fibres of the *m*. *temporalis profundus* are directed ventrally to insert at the base on the medial surface of the coronoid process. The lacrimal gland partially separates the *m*. *temporalis profundus* from the *m*. *temporalis posterior*. The nervous, vascular and glandular tissue of the optic channel separates the origins of *m*. *temporalis profundus* from the superficial part of the *m*. *pterygoideus medialis* (Fig. 1F).

#### Pterygoid complex

**Fig 6:**
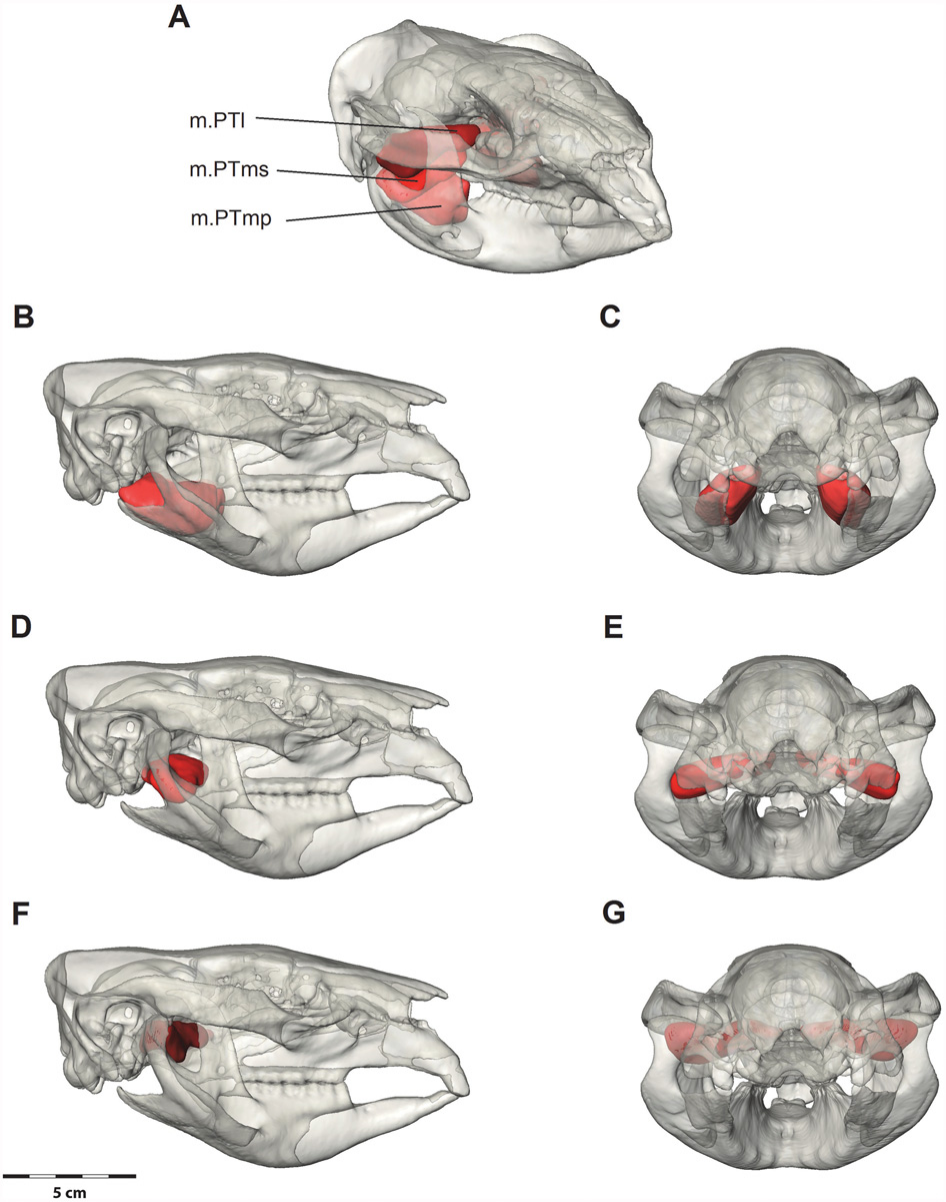
Digital dissection of the pterygoid muscle group. (A) Oblique view with all pterygoid muscles; (B, C) deep medial pterygoid (m.PTmp) in lateral and posterior views; (D, E) superficial medial pterygoid (m.PTms); (F, G) lateral pterygoid (m.PTl). Bone is at 70% transparency.

The pterygoid complex (Fig. 6) is smaller than the masseter and temporalis muscles (Table 1). It has a wide, mostly fleshy origin on the lateral pterygoid, sphenoidal pterapophysis, palatal and alisphenoid bones, and an insertion onto the medial surface of the mandible, the dorsal angular process, medial edge of the condylar neck and medial one third of the anterior surface of the condylar disk.

*M*. *pterygoideus medialis* (m.PTm) has been greatly enlarged by the inflected angle of the mandible and is divided into two parts: a deep part and a superficial part. The superficial part (m.PTms) originates from the deep pocket on the lateral surface of the alisphenoid formed by the descending process and lateral wing of the pterygoid bone. It inserts into the expansive pterygoid fossa formed by the inflected angle of the mandible, and on the medial surface of the ascending ramus, inferior to the insertion of the deep temporalis. The fibres of the superficial medial pterygoid pass ventrolaterally. The deep part (m.PTmp) originates on the narrow ventral edge of the pterygoid bone and inserts on the dorsomedial edge of the inflected angle of the mandible. Even though the deep part attachment points are narrow, the muscle has a large cross sectional area as its belly forms a broad compact mass between its attachments. Separating the insertions of each part of the medial pterygoid is nervous, vascular and glandular tissue emanating from the mandibular foramen.

*M*. *pterygoideus lateralis* (m.PTl) is a short, tubular muscle that originates from the lateral surface of the squamosal, medially to the glenoid and above the origin of the superficial medial pterygoid. The fibres run posterolaterally to insert on the medial aspect of the condylar disc of the temporomandibular joint. The lateral pterygoid has a small cross-sectional area.

### Comparison of methods and limitations

Two methods were used to estimate the proportion that each muscle contributes to the entire muscle system: wet weights from dissection, and volume from 3D “virtual dissection” using MRI. Both methods produce a similar estimate for the temporalis group and masseter group for specimen 3, which was both dissected and scanned (Table 2). However, there is more variation between the individual muscle sections within each group. This is most likely due to the difficultly in dividing the muscle layers during dissection. When an incomplete fascia divided muscle sections, the muscle fibres are sliced along a trajectory following that fascia. When forming a 3D model from MRI data, the division between muscles is slightly clearer.

**Table 2:** A comparison between traditional dissection and digital dissection.

Muscle	Mean Weight (g)	Mean Weight (%)	Volume (mm^3^)	Volume (%)
m.Tpr	12.565	8	31907	12
m.Tpo	28.825	18	31391	12
m.Tl	4.85	3	11785	4
**Temporalis Total**	**46.24**	**29**	**75084**	**28**
m.ZMM	15.905	10	19524	7
m.Mp	12.365	8	35941	13
m.Msi	36.895	23	51374	19
m.Mse	26.81	17	42536	16
**Masseter Total**	**91.975**	**58**	**149375**	**55**
m.PTmp	8.565	5	20925	8
m.PTms	6.275	4	13765	5
m.PTl	4.43	3	11640	4
**Pterygoid Total**	**19.27**	**12**	**46330**	**17**
**Total Muscles**	**157.485**	**100**	**270790**	**100**

Mean muscle weights of the left and right side from the gross dissection of specimen 3, and muscle volumes from the MRI digital dissection of the same specimen, including percentages for each muscle.

The pterygoid muscle group has the largest variation between estimates. The dissection produced an estimate 30% less than the 3D digital dissection. This group is difficult to dissect due to its deep location and it is generally dissected last, contributing to desiccation of the muscles. This could result in an underestimate of muscle weight. The digital dissection allows the true dimensions and positions of each muscle to be easily visualised and quantified. Therefore, the estimate from the digital dissection may be more accurate. Another method that was not tested here is the collection of dry weights that could address this issue.

Similarly, the masseter muscle group is usually dissected first, which could contribute to the slightly higher estimate from the dissection. The interior superficial masseter has a higher estimated percent from the dissection compared with the MRI volume because the separation between it and the deep masseter is not clear. However, due to the wrap-around nature of the masseter, the MRI reconstruction may not be entirely accurate either. It is, however, encouraging that the total percentages for each muscle group provided by the two methods is reasonably close.

## Discussion

The largest jaw muscle group in the common wombat (*Vombatus ursinus*) is the masseter. Wombats have increased the mass of the masseter by developing a large insertion area via a flared masseteric crest and by expanding the zygomatic arch laterally and moving the molar tooth rows medially [2,4,7]. The interdigitated nature of the masseter, with numerous tendinous attachments at the origin and insertion, may provide additional strength to the muscle and provide a high bite force. The partial fascia that divides the masseter decrease fibre length and increase pennation of the muscle, thereby increasing muscle force. The short rostrum also decreases the out-lever of the masseter system, thus increasing bite force, and the dorsoventrally compressed skull also offers a greater lateral leverage. Coupled with ever-growing hypsodont molars and a highly ankylosed symphysis, these adaptations have evolved in response to the wombats tough, abrasive diet [7,22].

The anatomy of the opossum, often used as a basic comparative model, demonstrates that there are regions of interconnection between the three major mastication muscles. These connections unite the masseter with the zygomaticomandibularis and in turn, the zygomaticomandibularis to the temporalis [10]. This may provide coordination between the masseter and temporalis for the function of orthal biting action. We note that the connectedness between the masseter, zygomaticomandibularis and temporal muscles is present in *V*. *ursinus*, and that the masseter in particular is elaborated and partially divided by lamina. This converges on the condition in ungulates and artiodactyls in particular, where complex masseter laminations are well documented [8]. A greater degree of functional coordination may be required for herbivores that employ greater orthogonal movements in combination with orthal movements for grinding food.

The muscle proportions for the *V*. *ursinus* estimated in this study differ slightly from those reported for the hairy-nosed wombat (*Lasiorhinus latifrons*) [4]. The masseter is larger in *V*. *ursinus* compared to that in *L*. *latifrons* due to the deeper masseteric fossa [23]. This confirms the predictions by Nakajima and Townsend [5] that the temporalis muscles of *L*. *latifrons* is larger and better developed than that of *V*. *ursinus* based on the wider, and rougher attachment area. This may provide an overall higher bite force in *L*. *latifrons* than *V*. *ursinus*.

According to Murray [7], mastication in wombats consists of a high rate of powerful chewing strokes, consisting of high compressive forces maintained during modest horizontal excursions. Generally, horizontal movement of the mandible is performed by the masseter muscle, while vertical movement is performed by the temporalis. Based on this study, the larger masseter muscles in *V*. *ursinus* may be associated with wider horizontal movements of the mandible during mastication compared to that in *L*. *latifrons*. This may be supported by the presence of wear facets on the incisors of the two genera, where *V*. *ursinus* is horizontal and *L*. *latifrons* is vertical and posterior [23]. Both species of wombat prefer green grass, and will only consume roots and bark when green grass and surface water are unavailable [22]. *V*. *ursinus* inhabits temperate forests and wetter regions in New South Wales, Victoria and Tasmania, while *L*. *latifrons* inhabits arid regions of South Australia and rarely has access to water, especially in summer [1]. Therefore, *L*. *latifrons* grazes on higher fibre vegetation, that is lower in water and protein, and consume more roots and bark than *V*. *ursinus* [22,24]. It is suggested that *L*. *latifrons* may require a stronger temporalis, and higher bite force compared to *V*. *ursinus*, due to the drier and tougher vegetation it consumes. The significance of the difference in muscle proportions between wombat genera may be confirmed or denied with a greater sample size.

The masticatory apparatus of wombats has been described as being similar to rodents, specifically sciuromorph rodents (squirrel-like) [7]. Similarities with rodents include the enlarged, single pair of upper incisors, the presence of a diastema, the lack of a masseteric process, a deeply excavated maxillojugal region, and hypsodont cheek teeth arranged close to the midsagittal axis of the cranium. However, the symphysis of the lower jaw in wombats is firmly fused, unlike that in the majority of rodents, where it is mobile. Further differences include the wombat’s large inflected mandibular angle, the enlarged medial pterygoid muscle, the narrow transversely oriented mandibular condyle, and the position of enamel on its molars. Functionally, the masticatory complex also differs between wombats and rodents. Rodents exhibit two discrete modes of feeding—gnawing at the incisors and chewing at the molars—with propalinal movement of the lower jaw between the two [13,25]. These movements are necessary for rodents because the incisors and molars do not occlude simultaneously as they do in wombats. In wombats, this movement it restricted by strong ligaments at the temporomandibular joint. While there are many similarities with rodents, the differences reflect the ability of wombats to exert very high compressive forces on the tooth row simultaneously with the horizontal movement of the mandible.

### Variation in Vombatomorphia

Taxa in the Vombatomorphia Aplin & Archer, 1987 [26] are well known for exhibiting a high degree of individual variation, often considered to be close to the polar extremes for variation within morphospecies [27]. The variability within wild populations of wombats (all three species) is no exception. For example, Merriless [28] found abnormalities, pronounced asymmetries and supernumerary teeth in more than half the specimens sampled. Cranial characters are sufficient to separate the modern species at the generic level, but Merriless [28] cautioned against the minutiae of characters often used to distinguish fossil wombat taxa. Crowcroft [29] and Scott et al. [23] re-evaluated diagnostic for wombat genera and Dawson [30] supported the division made by Tate [31] of *V*. *ursinus* into three extant subspecies and one extinct subspecies, with a further three extinct *Phascolomys* species synonomised with *V*. *ursinus*. Several authors state that *V*. *ursinus* females tend to be larger than males in general body dimensions and weights [6,32], and for cranial measures [5], but these are not statistically significant within the total variation. Green and Rainbird [33,34] report no dimorphism in the *V*. *ursinus* populations of northern Tasmania. Given the large degree of individual variation between wombats, we caution the reader about the variance to be expected beyond the limited sample results of the dissections we report here.

### Digital dissections

Three-dimensional (3D) digital dissections provide a valuable resource to identify, visualise and document the muscular anatomy in extant species [13,15–17]. One of its main advantages over gross dissection is that soft-tissue anatomy can be investigated *in situ*, allowing a greater understanding of their complex spatial arrangement in three dimensions. When performed prior to gross dissection, digital dissections can also be used to provide guidelines for the physical dissection process. For example, many fascia divide the masseter muscles and these can be visualised from the MRI so that the major muscle bundles can be more clearly identified during the gross dissection. As a non-destructive method, we also have the ability to return to the digital dissections and confirm or correlate findings after the gross dissection.

Digital dissections also allow us to visualise and measure the true dimensions of soft-tissues by segmenting the individual structures. This provides a means to obtain quantitative measurements of volume, fibre length and cross-sectional areas that can be used for biomechanical studies. These measurements are potentially more accurate compared to quantitative data from gross dissections that can be affected by desiccation of the soft-tissue. As this study shows, deep muscles like the pterygoids that are often dissected last can lose a significant amount of moisture, providing a smaller estimate. This limitation in fresh dissection could be addressed by drying the muscles before collecting the weight. However, this data may not be comparable with other literature presenting wet weights. Blunt dissection through muscle bundles can also affect the measurements of muscle weights, especially for the wombat masseter with its interdigitated nature where deeper layers are fused to form a continuous muscle mass. However, more comparisons between digital and gross dissections would be needed to know the full extent of the variation.

Although MRI scans can provide a significant increase in our understanding of the spatial arrangement of internal features in relation to other soft and hard tissues, there are still some limitations when performing digital dissections. CT and MRI scanning come with monetary costs, and the digital visualisation and segmentation of the scan data often requires expensive software and hardware. The manual segmentation of specific organs and individual soft-tissue structures can also be time consuming and requires a considerable degree of technical expertise and training in the software package. Furthermore, while it can be relatively accurate to quantify muscle volume and cross-section area, other properties, such as pennation angle and fascicle length cannot be measured from MRI scans. A method of resolving some of these limitations in visualisation is the process of iodine staining and micro-CT scanning [12,13,15,17]. The iodine staining provides contrast and clarity of soft-tissue anatomy more vividly than MRI. The clarity in the scans then allows visualisation and measurement of individual muscle fibres which can be used to measure muscle force [13,35,36].

## Conclusions

Wombat jaw musculature is three-dimensionally complex and difficult to illustrate in 2D. Previously, the soft tissue anatomy of the Vombatidae has been based on *Lasiorhinus latifrons*. This study of the common wombat, *Vombatus ursinus* masticatory muscles is the first detailed visual documentation and description for this species. The masticatory system of *V*. *ursinus* was found to reflect minor differences in soft tissue structure and function, that had been predicted based on the bony morphology of *V*. *ursinus* and *L*. *latifrons*. Additionally, we provide the first volumetric model of wombat musculature based on CT and MRI data for *V*. *ursinus*. The downloadable, 3D model of the wombat jaw muscles is available as a tool for morphologists, biologists, and the general public. It is also useful as a reference for the reconstruction of soft tissue in related extinct marsupials, such as those within the Diprotodontidae and Palorchestidae [7,37].

## Supporting Information

S1 Fig Interactive 3D PDF showing the digitally segmented cranium, mandible and jaw-closing muscles of *Vombatus ursinus*. Simplified 3D model of the skull and jaw muscles of *Vombatus ursinus*. If viewing in Adobe Reader, click the figure to activate the interactivity. To visualise the model, use the zoom, pan and rotate functions, change the background colour, use the standard views and toggle on and off parts in the model tree. Parts can also be made transparent.
(PDF)

S1 Movie Computed tomography (CT) slices of *V*. *ursinus* in frontal view. (MPG)

S2 Movie Magnetic Resonance Imaging (MRI) slices of V. ursinus in frontal view. (MPG)
