# Supplementary material for: Morphology of the Jaw-Closing Musculature in the Common Wombat (Vombatus ursinus) Using Digital Dissection and Magnetic Resonance Imaging
Source: PLoS One. 2015 Feb 23;10(2):e0117730. doi: 10.1371/journal.pone.0117730 (PMC4338273; doi:10.1371/journal.pone.0117730)
Supplement: S1 Fig — Simplified 3D model of the skull and jaw muscles of Vombatus ursinus. If viewing in Adobe Reader, click the figure to activate the interactivity. To visualise the model, use the zoom, pan and rotate functions, change the background colour, use the standard views and toggle on and off parts in the model tree. Parts can also be made transparent. (PDF) [file pone.0117730.s001.pdf]

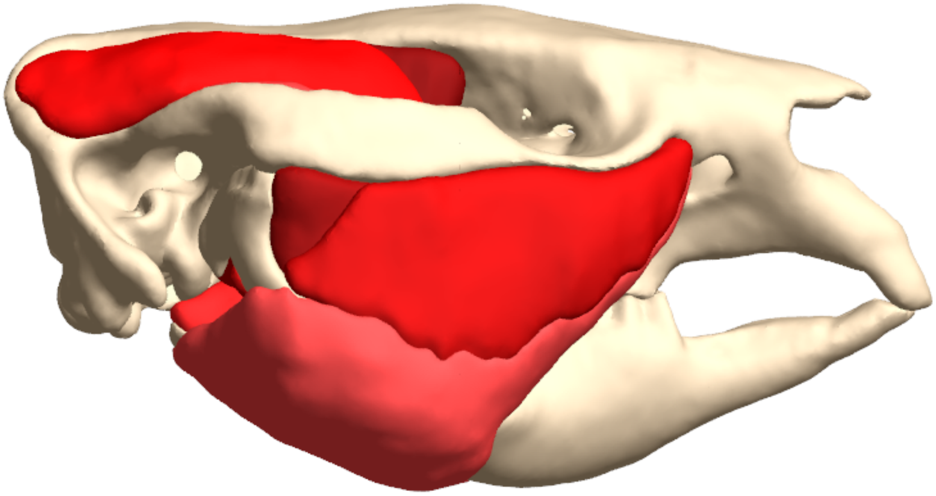

Figure S1 Interactive 3D PDF showing the digitally segmented cranium, mandible and jaw-closing muscles of *Vombatus ursinus*. Simplified 3D model of the skull and jaw muscles of *Vombatus ursinus*. If viewing in Adobe Reader, click the figure to activate the interactivity. To visualise the model, use the zoom, pan and rotate functions, change the background colour, use the standard views and toggle on and off parts in the model tree. Parts can also be made transparent.
